# Domestication, breeding, omics research, and important genes of *Zizania latifolia* and *Zizania palustris*

**DOI:** 10.3389/fpls.2023.1183739

**Published:** 2023-05-31

**Authors:** Yan-Ning Xie, Qian-Qian Qi, Wan-Hong Li, Ya-Li Li, Yu Zhang, Hui-Mei Wang, Ya-Fen Zhang, Zi-Hong Ye, De-Ping Guo, Qian Qian, Zhong-Feng Zhang, Ning Yan

**Affiliations:** ^1^ Tobacco Research Institute of Chinese Academy of Agricultural Sciences, Qingdao, China; ^2^ State Key Laboratory of Rice Biology, China National Rice Research Institute, Hangzhou, China; ^3^ Zhejiang Provincial Key Laboratory of Biometrology and Inspection and Quarantine, College of Life Sciences, China Jiliang University, Hangzhou, China; ^4^ Department of Horticulture, College of Agriculture and Biotechnology, Zhejiang University, Hangzhou, China

**Keywords:** wild rice (*Zizania* spp.), edible history, economic value, domestication process, breeding objectives, omics research, important genes

## Abstract

Wild rice (*Zizania* spp.), an aquatic grass belonging to the subfamily Gramineae, has a high economic value. *Zizania* provides food (such as grains and vegetables), a habitat for wild animals, and paper-making pulps, possesses certain medicinal values, and helps control water eutrophication. *Zizania* is an ideal resource for expanding and enriching a rice breeding gene bank to naturally preserve valuable characteristics lost during domestication. With the *Z. latifolia* and *Z. palustris* genomes completely sequenced, fundamental achievements have been made toward understanding the origin and domestication, as well as the genetic basis of important agronomic traits of this genus, substantially accelerating the domestication of this wild plant. The present review summarizes the research results on the edible history, economic value, domestication, breeding, omics research, and important genes of *Z. latifolia* and *Z. palustris* over the past decades. These findings broaden the collective understanding of *Zizania* domestication and breeding, furthering human domestication, improvement, and long-term sustainability of wild plant cultivation.

## 1 Introduction

The genus *Zizania* (family Poaceae) is a type of wild rice that includes four species: *Z. aquatica*, *Z. palustris*, *Z. texana*, and *Z. latifolia* (**Table 1**) (Makela et al., 1998; Lu et al., 2022). *Zizania* diverged from *Oryza* approximately 26–30 million years ago (mya), whereas *Z. palustris* and *Z. latifolia* diverged from one another approximately 6–8 mya (Haas et al., 2021). Due to the differences in geographic distribution and ecological environments, a significant variability has been observed between the morphology and reproductive cycle of species in East Asia (*Z. latifolia*) and those in North America (*Z. aquatica*, *Z. palustris*, and *Z. texana*). *Z. aquatica* is found in Southern Ontario, Quebec, the East Coast of Canada, and the Atlantic and Gulf Coasts of the US (Florida and Louisiana) (Xu et al., 2010). *Z. palustris* is widely distributed across the Canadian prairie provinces (Alberta, Manitoba, and Saskatchewan), along the Great Lakes region, and across the prairies in North America (Mcgilp et al., 2020; Haas et al., 2021). *Z. texana*, a rare species of *Zizania*, is limited to a small region along the San Marcos River in Texas. Its small range and limited population size have led to it being classified as endangered by the US federal government (Poole and Bowles, 1999; Richards et al., 2004; Tolley-Jordan and Power, 2007). In numerous places along the Great Lakes, areas with abundant *Zizania* populations are already endangered. The growing body of research on *Zizania*, including genetics, domestication, breeding, etc., has greatly enhanced our ability to conserve this important plant species (Kennard et al., 2002; Lu et al., 2005; Kahler et al., 2014; Haas et al., 2021). The morphology of East Asian wild rice differs from its North American counterpart (**Table 1**). *Z. latifolia*, also known as East Asian wild rice, is believed to have originated in China and is found in various water bodies including rivers, lakes, ditches, ponds, and paddy fields throughout the country, especially in the Yangtze and Huaihe River basins (Yan et al., 2018). This species is not limited to China and can also be found in other parts of East Asia, such as Japan, South Korea, and Southeast Asia (Fan et al., 2016; Chen et al., 2017; Yan et al., 2018).

**Table 1:** Comparison of basic information of *Zizania*.

Type	Distribution	Growth type	Chromosome number	Industrial/Cash crop	Harvesting method	Reference
*Z. aquatica*	Canada and the United States	Annual	2n = 2× = 30	No	Artificial picking	Xu et al., 2010
*Z. palustris*	Canada’s coastal provinces and the surrounding areas of the Great Lakes in North America	Annual	2n = 2× = 30	Yes	Mechanical harvesting	Mcgilp et al., 2020; Haas et al., 2021
*Z. texana*	Along the San Marcos River in Texas, USA	Perennial	2n = 2× = 30	No	Artificial picking	Poole and Bowles, 1999; Richards et al., 2004; Tolley-Jordan and Power, 2007
*Z. latifolia*	China, Japan, South Korea	Perennial	2n = 2× = 34	Yes	Artificial picking	Fan et al., 2016; Chen et al., 2017; Yan et al., 2022

Wild rice, classified as a whole grain, is a caryopsis with a seed coat that is thinner and longer in shape than conventional rice (*Oryza sativa*). The grain has acuminose ends, black-brown and glossy cortex, and a milky white and brittle endosperm (Surendiran et al., 2014). Zizania is a highly nutritious food, rich in protein, dietary fiber, vitamins, and minerals, with a low glycemic index, low fat content, and reasonable amino acid composition (Zhai et al., 2001; Yu et al., 2020). Additionally, wild rice contains various biologically active substances such as phytosterol, γ-oryzanol, γ-aminobutyric acid, and phenolic compounds, which have potential health benefits (Yu et al., 2022). Several studies have shown that whole grain consumption helps reduce the risk of chronic diseases, such as cardiovascular disease, obesity, cancer, and diabetes (Juan et al., 2017). Various international projects have been initiated in the 21^st^ century aimed at increasing the benefits and content of plant functional components in whole grain for lowering cholesterol, and regulating blood glucose metabolism in dietary fiber, in addition to reducing the risk of cardiovascular and cerebrovascular diseases (Ward et al., 2008; Juan et al., 2017; Guo et al., 2022).

Northern wild rice (NWR, *Z. palustris*) serves as a traditional food among American Indians and has gradually been incorporated into the diets of various cultures across the world (Qiu et al., 2010). Following its “nonshattering” domestication in the late 1960s, *Z. palustris* gained extremely high commercial value. Currently, large-scale commercial planting of *Z. palustris* is concentrated primarily in the US (Minnesota and California) (Mcgilp et al., 2020). *Z. latifolia* has a long history in ancient China; however, due to the human population increases in Southern China following the Tang and Song Dynasties, subsequent agricultural development, and the man-made drying of lakes for rice cultivation, the prevalence of *Z. latifolia* has drastically reduced (Yan et al., 2018). Moreover, the low yield of *Z. latifolia* caused by the difficulty in its harvesting (seeds are prone to fall off after ripening) is another contributing factor. In addition, with the widespread cultivation of rice, the yield of rice has increased, reducing the importance of human reliance on Chinese wild rice (CWR) as a staple food. Therefore, CWR has gradually faded out of people’s lives (Yan et al., 2018). In contrast, wild rice remains an expensive and popular food in North America (Surendiran et al., 2014), while it is not often consumed in modern China.

The global population is expected to exceed 9 billion by 2050; thus, higher agricultural output is required to meet the growing demand for food. However, the acceleration of urbanization and reduction of resources (land, water, and human resources available for agriculture) have presented major challenges to production requirements (Zhang et al., 2022). For example, *Z. latifolia*, a known perennial crop that does not require replanting after each harvest, unlike annuals such as *Z. palustris*, whose cultivation mandates substantial seed input, increased investment in agricultural machinery, greater water and soil loss, soil nutrient depletion, as well as other socio-economic and ecological challenges (Lobell et al., 2011). Therefore, breeding perennial food crops (e.g., *Z. latifolia*) can serve as a novel approach to tackling food security and environmental challenges (Glover et al., 2010). *Zizania* breeding and domestication have recently become a necessary direction in large-scale commercial farming and can bring favorable economic gains to farmers. Currently, genomics approaches are widely used for crops such as rice and have provided new directions for crop domestication and utilization of heterosis (Huang et al., 2012; Huang et al., 2016; Yu and Li, 2022). In particular, the multi-omics methods, including genomics, transcriptomics, proteomics, and metabolomics, have been successfully utilized for the genetic improvement of crops, achieving more efficient and accurate breeding through molecule design (Zeng et al., 2017; Wu et al., 2021). This article reviews the research progress on the edible history, economic value, domestication, breeding, omics research, and functional verification of important genes of *Z. latifolia* and *Z. palustris* over the past few decades, providing strong theoretical support for greater domestication of this genus with high-yield, -quality, -stress resistance, and -nutrient utilization.

## 2 Edible history and economic value of *Z. latifolia and Z. palustris*

**Figure 1:**
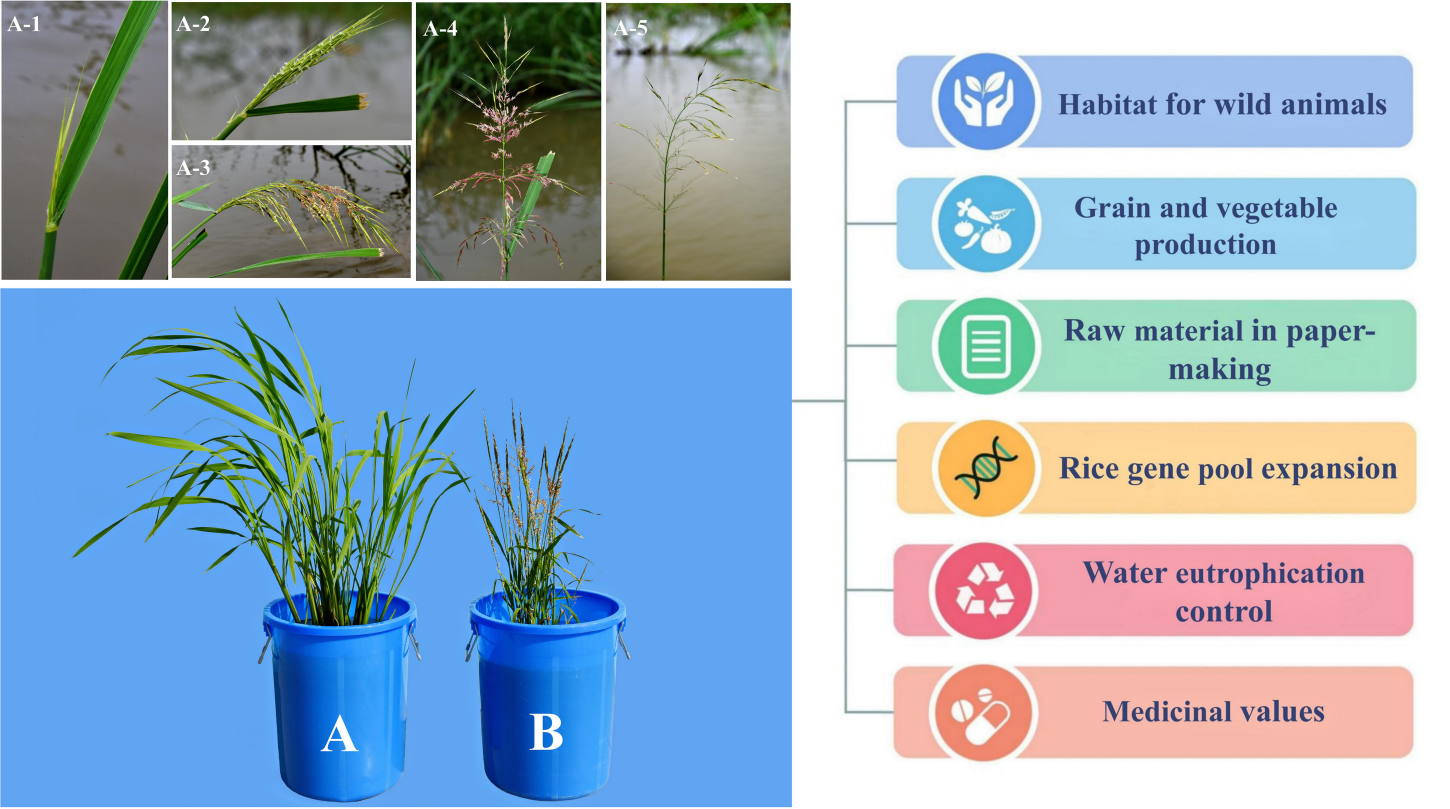
Application value of *Zizania latifolia* **(A)** and *Zizania palustris* **(B)**. A1–A5 represent the inflorescences of *Z. latifolia* at different stages after flowering.

*Zizania* spp., particularly *Z. palustris* and *Z. latifolia*, have extremely high ecological and economic values, as they provide habitat for wild animals, in addition to their use as grains and vegetables. Furthermore, wild rice is used as a raw material in paper-making and a genetic resource for expanding the rice gene pool; it is also used in controlling water eutrophication and possesses certain medicinal values (**Figure 1**).

### 2.1 Edible history and economic value of *Z. palustris*

*Z. palustris*, also known as wild rice, is an annual, outcrossing, aquatic species in the Poaceae family and native to North America (Hayes et al., 1989; Zaitchik et al., 2000; Kennard et al., 2002). Currently, *Z. palustris*, a nutritious grain, is the only successfully domesticated and widely planted wild rice. *Z. palustris* is typically found in shallow lakes, rivers, and coastal areas within the north-central US and southern Canada (Oelke, 2004). This plant provides habitats for various birds, mammals, fish, and invertebrates. In addition, *Z. palustris* participates in the water nutrient cycle and helps stabilize the sediment of coastal river wetlands (Meeker, 1996), playing a significant role in the local food network and wetland ecology (Drewes and Silbernagel, 2012). For centuries, American Indians have collected *Z. palustris* from the lakes and rivers of the Great Lakes region (Hayes et al., 1989). *Z. palustris* is considered a high-value crop and has become a cash crop in recent years. *Z. palustris* is classified as a whole grain food containing > 75% carbohydrates, 6.2% dietary fiber, 14.7% protein, and 1.1% lipids (Yan et al., 2018; Yu et al., 2021). In addition, the antioxidant activity of *Z. palustris* is 10–15 times higher than that of rice, while its protein and essential amino acid content are doubled, and dietary fiber content is 5 times greater, with a low-fat content, most of which are essential unsaturated fatty acids, including ω-6 (35.0–37.8%) and ω-3 (20.0–31.5%) (Wiser, 1975; Bunzel et al., 2002; Aladedunye et al., 2013; Kahler et al., 2014; Surendiran et al., 2014; Timm and Slavin, 2014). History has documented the economic position of wild rice. Early Native Americans, especially the Ojibway, Menomini, and Cree peoples, consider *Z. palustris* a traditional food (Lorenz and Lund, 1981). In the 17^th^ century, Europeans successively entered the Great Lakes Region of North America, where a priest named Hennepin was the first to observe the collection of *Z. palustris* by American Indians, describing it as an abundant aquatic oat that grows in the lakes without any cultivation (Steeves, 1952). Hennepin believed that aquatic oats were an inadequate translation and revised it to wild rice (Horne and Kahn, 1997).

Currently, the indigenous populations hand-harvest and sell *Z. palustris*, representing an important component of their income (Drewes and Silbernagel, 2012). In northern Michigan and Wisconsin, as well as most of northeastern Minnesota, tribes have preserved their methods of *Z. palustris* processing, a right protected by law (Walker et al., 2006). Although American Indians have processed and harvested *Z. palustris* for food over many centuries, researchers first proposed the domestication of *Z. palustris* as a cultivated crop in the mid-18th century (Porter, 2019). However, in the 1950s, paddy field planting and harvesting of *Z. palustris* began in earnest (Porter, 2019). *Z. palustris* seeds are large and can produce considerable yield, being highest in Minnesota and California (Oelke, 2004). However, this crop has relatively fragile seeds and inconsistent ripening periods, making it difficult to achieve full domestication (Oelke and Albrecht, 1978; Drewes and Silbernagel, 2012). Currently, Canada, Hungary, and Australia have implemented commercialized production of *Z. palustris* (Qiu et al., 2009; Small, 2012); however, *Z. aquatica* and *Z. texana* seeds are fragile, have low yields, while their distribution ranges and population sizes are substantially smaller than that of *Z. palustris*, restricting their collection for consumption. Moreover, relatively little research and domestication efforts have been dedicated to these species (Oxley et al., 2008).

### 2.2 Edible history and economic value of *Z. latifolia*

The history of harvesting and utilizing wild rice by Chinese ancestors dates to the Zhou Dynasty 3000 years ago (Yan et al., 2018). Wild rice was a precious food material offered to royalty in Ancient China, and its historical position exceeds that of other crops (Yan et al., 2018). The economic value of *Z. latifolia* primarily rests on two aspects: CWR is a nutritious whole-grain food that is being increasingly accepted, and *Z. latifolia* is the source of the domesticated, cultivated species *Jiaobai* (Yu et al., 2020). Approximately 2000 years ago, *Z. latifolia* infected by *U. esculenta* resulted in culm enlargement and the formation of a fleshy edible gall—*Jiaobai*. Based on this process, Chinese ancestors domesticated it into a vegetable (Zhao et al., 2018; Tu et al., 2019). *Jiaobai* is a famous aquatic vegetable that has also been introduced to Japan, South Korea, and across Southeast Asia (Xu et al., 2010). Currently, the total planting area of *Jiaobai* in China is > 60,000 hm^2^, second only to lotus as the most cultivated aquatic vegetable in China (Yan et al., 2018). The cultivation of this vegetable is an important economic resource for many households in Southern China (Guo et al., 2007; Yan et al., 2013a). *Jiaobai* is a widely enjoyed fresh, tender, crisp, and sweet vegetable that provides sugar, protein, vitamins, minerals, and essential amino acids (Yan et al., 2018). In addition, enzyme-treated *Z. latifolia* extract (ETZL) can clear free radicals and block elevated triglyceride and malondialdehyde levels in the liver (Chang et al., 2021). Moreover, ETZL significantly decreases t-BHP-induced HepG2 cytotoxicity and active oxygen generation and improves liver damage induced by excessive alcohol consumption via upregulating antioxidant defense mechanisms to prevent alcoholism (Chang et al., 2021; Gao et al., 2022). ETZL and its main compound, tricin, can inhibit the production of metalloproteinases in the extracellular matrix of human skin following UV exposure, thereby helping the skin resist ultraviolet radiation (Park et al., 2019).

The caryopsis of *Z. latifolia* is classified as one of the “six grains”, along with rice, broomcorn millet, panicled millet, wheat, and beans, in Ancient China. As a grain, *Z. latifolia* primarily exists in a wild state and has not been artificially domesticated (Yan et al., 2022). Although the edible history of *Z. latifolia* traces back to the Zhou Dynasty (3 kya), *Z. latifolia* was gradually replaced by cultivated rice due to its strong seed shattering and low yield characteristics (Yan et al., 2022). *Z. latifolia* was subsequently used as a traditional Chinese medicinal crop that was categorized as a treatment for diabetes and gastrointestinal diseases in the Compendium of Materia Medica of Li Shizhen during the Ming Dynasty (Yu et al., 2022). *Z. latifolia* is classified as a whole grain with high nutritional value. In particular, it is rich in protein, essential amino acids, fatty acids, vitamins, and microelements (Yan et al., 2018). Due to its high nutritional value, developing germplasms resistant to seed shattering remains a top priority in the domestication and breeding of *Z. latifolia* (Xie et al., 2022). As a graminaceous crop closely related to rice, *Z. latifolia* boasts myriad beneficial traits that rice lacks, including rice blast resistance, thick stalks, strong tillering, low-temperature resistance, flood resistance, rapid grouting maturity, high biological yields, and optimizing protein and lysine contents in seeds (Chen et al., 2012; Ye et al., 2016; Yan et al., 2022). Hence, it is capable of overcoming the narrow bottleneck of genetic resources for rice breeding and providing important materials for optimizing genetic traits. For example, a *Z. latifolia* and rice hybrid exhibited increased rice blast resistance (Wang et al., 2013). However, the genomes of hybrid rice strains comprise a small proportion of *Z. latifolia* DNA sequences, and inserting exogenous DNA fragments introduces extensive cytosine methylation variation and transposon activation. The resulting sequence variation may serve as the primary driver of character variations within introgressive hybrid lines (Liu et al., 2004; Shan et al., 2005; Wang et al., 2005). Meanwhile, given the advantageous characteristics of *Z. latifolia*, it may represent a source of genes for future rice molecular breeding. As such, the introduction of *Z. latifolia* as a perennial food crop may prove ecologically beneficial and sustainable, thus providing a means to address food security and environmental challenges. Indeed, appropriate domestication of *Z. latifolia* across large planting areas can fulfill the demand of an increasing population for a nutritional food source while alleviating the environmental challenges driven by the reduction of cultivated land and resources.

Previous studies have shown that *Z. latifolia* contains phytoliths, showing considerable carbon sequestration potential (Li et al., 2022). In addition, this species exhibits excellent sequestration under nitrogen and phosphorus eutrophication conditions compared to other plants. Planting *Z. latifolia* in highly eutrophicated areas with seasonal harvesting and clearing may effectively control pollution through plant absorption and microbial degradation (Liu et al., 2007; Tang et al., 2013). During industrial production, *Z. latifolia* has found further application as pulp for paper making, which can be prepared by sulfate treatment of the residual stems and leaves after *Jiaobai* harvesting. Physical performance evaluations have shown that the tear, tensile, and rupture strengths of the resulting paper are not significantly different from those of conventional old corrugated container paper (Chen et al., 2022).

## 3 Domestication and breeding of *Z. latifolia and Z. palustris*

Crop domestication is the foundation of modern agriculture and comprises long and complex evolutionary processes (Pickersgill, 2007; Vaughan et al., 2007). Improving the cultivation of wild plants entails changing their morphology and physiology to accommodate human and production needs, which is fundamental to agricultural development (Tang et al., 2013). Although the current growth of global crop yield is stable, botanists face an enormous challenge of food security in addition to climate change and the increasing growth of the global population (Ren et al., 2005; Ma et al., 2015).

### 3.1 Domestication and breeding of *Z. palustris*

Compared to rice, maize, wheat, and other major food crops, *Z. palustris* has a shorter breeding and commercial production time. Hundreds of years ago, American Indians processed and harvested *Z. palustris* for food; however, only in 1853, *Z. palustris* was proposed as a crop for planting (Porter, 2019). In 1950, farmers in Northern Minnesota successfully harvested the first batch of manually planted *Z. palustris*. In 1962, Uncle Ben’s company signed contracts to purchase and sell *Z. palustris* with planters in Minnesota. In 1972, farmers planted *Z. palustris* in a paddy field; however, they could not harvest seeds due to its strong seed-shattering tendencies. *Z. palustris* planters consulted the University of Minnesota Agronomy and Phytogenetics Center for improvements, pursuing a cultivar suitable for large-scale planting. Accordingly, the University of Minnesota developed a special scientific research team to conduct the artificial domestication of *Z. palustris*, which has been ongoing for 40 years until now (Kahler et al., 2014). Currently, the primary objective of breeders for *Z. palustris* domestication is the improvement of various growth traits, including reducing seed shattering following maturation and increasing the seed maturation consistency (Grombacher et al., 1996). Seed shattering is an important trait of wild plants to adapt to natural environments and maintain reproduction levels (Zhang et al., 2009); further, it is considered a direct morphological basis for identifying the domestication of wild plants (Ray and Chakraborty, 2018). Although strong seed shattering facilitates self-breeding and population conservation of *Z. palustris*, the reduction of seed shattering can promote an effective collection of seeds after maturation, thus sustaining production and yield (Yan et al., 2015; Lv et al., 2018). During *Z. palustris* planting, excessive seed shattering can cause harvest loss within 24 h amounting to 10–20% of the total harvest, whereas the loss of seed maturity can reach 70% during the harvest season (Elliott and Perlinger, 1977; Kennard et al., 2002). Recent collinearity analyses of the *Z. palustris* and rice genomes screened 20 orthologous genes with rice seed shattering genes *qSH1*, *SH1*, *SHAT1*, *SH4*, *SH5*, and *OsLG1* as potential candidate genes to improve seed shattering (Haas et al., 2021).

A major component of the University of Minnesota *Z. palustris* breeding program centers around using conventional breeding methods for *Z. palustris*, selecting superior phenotypes that can significantly improve variety yield and production efficiency (Grombacher et al., 1996). Progress in crop improvement has been successful using the phenotypic mass recurrent selection method of plant breeding and has led to the introduction of improved wild rice varieties (Kennard et al., 2000). Certain *Z. palustris* hybrids are currently under commercial cultivation, among which “Itasca-C12” was released in 2007 by the Minnesota Cultivated Wild Rice Council as a variety with lower seed shattering that is suitable for mechanized harvesting in large planting areas. Therefore, “Itasca-C12” is a standard in the *Z. palustris* industry and is used for genome sequencing (Haas et al., 2021) and research on seed dormancy (Mcgilp et al., 2022) of *Z. palustris*. These artificially selected *Z. palustris* varieties are primarily grown in Minnesota and California (Haas et al., 2021). *Z. palustris* seeds have moderate tolerance to recalcitrance and dehydration, limiting their survival in off-site storage to 1–2 years. Hence, save for annual seed maintenance, *Z. palustris* cannot be stored in seed banks or repositories, posing challenges to *Z. palustris* conservation and breeding plans (Mcgilp et al., 2022). Seed dormancy refers to the lack of germination under generally favorable environmental conditions, i.e., sufficient water, oxygen, temperature, and light (Hilhorst, 1995). *Z. palustris* exhibits a minimum three-month dormancy period (Cardwell, 1978), which is affected by temperature, as well as the stability of seed pericarps and phytohormones, such as abscisic acid and gibberellic acid (Grombacher et al., 1996). Within Poaceae, 98.6% of species are classified as orthodox or desiccation tolerant, while only 0.8% and 0.6% are considered recalcitrant and intermediate, respectively (Dickie and Pritchard, 2002). *Z. palustris* is generally considered recalcitrant; however, its water resistance is impacted by storage temperature, degree of dryness, and metabolic activity (Probert and Longley, 1989; Kovach and Bradford, 1992; Berjak and Pammenter, 2008). *Z. palustris* seeds can survive drying under specific conditions, with embryonic water content reaching as low as 6% (Kovach and Bradford, 1992). Therefore, maintaining the vitality of wild rice seeds is essential for *Z. palustris* breeding (Kennard et al., 1999; Jin et al., 2005; Porter, 2019).

### 3.2 Domestication and breeding of *Z. latifolia*

**Figure 2:**
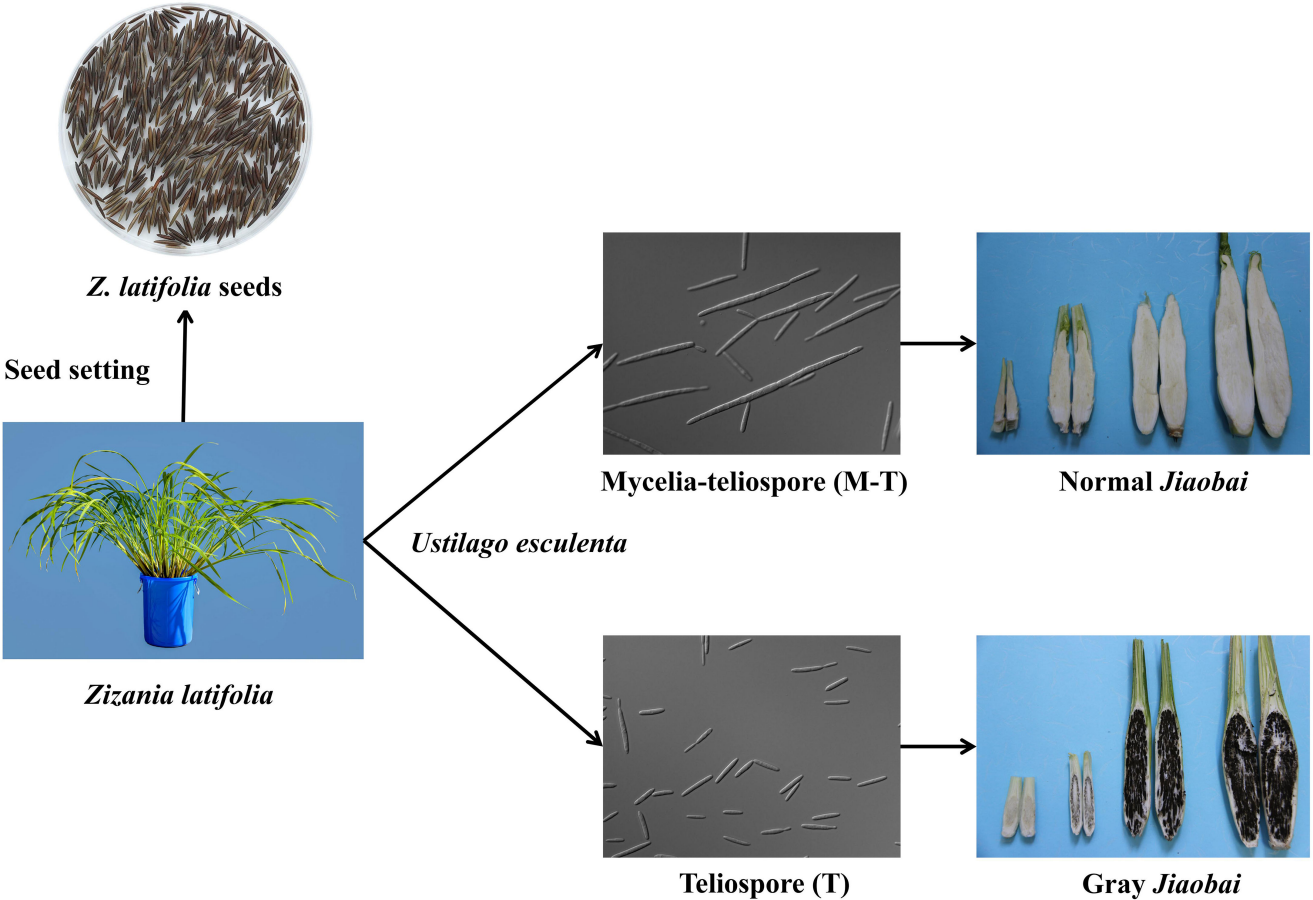
*Jiaobai* (normal and gray *Jiaobai*) formed from the infection of *Zizania latifolia* with *Ustilago esculenta*. In the images, *U. esculenta* is a haploid strain, the mycelia-teliospore (M-T) has a long haploid phase with multifocal budding, while the teliospore (T) has a short haploid phase with normal budding and generally does not exhibit multifocal budding.

The economic value of *Z. latifolia* is associated primarily with the aquatic vegetable *Jiaobai* and CWR (Yan et al., 2018). Chinese ancestors domesticated *Z. latifolia* as an aquatic vegetable Jiaobai approximately 2000 years ago (as noted in the Chinese first dictionary book “Erya” in the Qin Dynasty, 207–221 BC) (Guo et al., 2007; Guo et al., 2015). During its long-term symbiosis with the endophytic fungus *U. esculenta*, domesticated *Z. latifolia Jiaobai* has lost its flower structures and sexual propagation ability, only undergoing asexual reproduction through underground rhizomes (Guo et al., 2015). *U. esculenta* primarily colonizes the underground root and rhizome of the *Jiaobai* plant, completing its whole life cycle within the host (Yan et al., 2013b; Jose et al., 2016). Currently, China has > 100 regional cultivars of *Jiaobai*, and it has become an important economic source for many rural households in southern China (Guo et al., 2007). Domesticated *Jiaobai* exhibits significant divergence from *Z. latifolia* based on plant type, means of reproduction, and swollen gall metamorphosis. Under natural conditions, the fleshy stem of the plant formed after infection by *U. esculenta*, some of them are smaller and full of black teliospores, called “gray *Jiaobai*” (**Figure 2**). *Jiaobai* cultivars are compact, vertical, have swollen, fleshy stems, and lack or only possess rudimentary teliospores (“normal *Jiaobai*”; Yan et al., 2013a). Notably, the genetic variation and relationships of *Z. latifolia* and *Jiaobai* varieties exhibit obvious population structures (Zhao et al., 2019). However, the levels of genetic variation within *Jiaobai* are extremely low, suggesting that *Jiaobai* arose from a single domestication (Xu et al., 2008; Zhao et al., 2019). The domestication of *Jiaobai* and the diversity of its cultivars are mainly affected by the genetic variation of *U. esculenta* (Zhao et al., 2019). Specifically, *U. esculenta* strains in normal and gray *Jiaobai* resulting from atavistic mutation exhibit differences in morphology and internal transcribed spacers, with marked differentiation in the morphology and genetics between the two forms (You et al., 2011).

## 4 Omics research of Z. latifolia and Z. palustris

### 4.1 Genome research of *Z. latifolia* and *Z. palustris*

A reference genome is the foundation of genomic and gene function research (Jansen et al., 2007; Moore et al., 2010). Alignment of molecular markers and reference genomes can provide researchers with genes of interest and functional relationships between characteristics, important physiological mechanisms, and the structures of intraspecies genetic diversity. However, as of 2020, the genome resources of *Z. palustris* were limited to only a few studies on molecular markers, including isozyme (Lu et al., 2005), restrictive fragment length polymorphism (Kennard et al., 1999; Kennard et al., 2002), simple sequence repeats (Kahler et al., 2014), and single nucleotide polymorphism (Shao et al., 2020). In 2021, Haas et al. (2021) assembled the high-quality genome of the ‘Itasca-C12’ NWR cultivar and anchored 98.53% of sequences to 15 chromosomes, with an assembly length of 1.29 Gb and high reproducibility (~76.0%; **Table 2**). This genome assembly and annotation provide an important reference to the comparative genomics of the rice tribe (Oryzeae) and lays a foundation for future NWR protection and breeding work (Haas et al., 2021). Estimates of divergence times revealed that the *Zizania* genus diverged from *Oryza* by approximately 26–30 mya, whereas *Z. palustris* and *Z. latifolia* diverged from one another by approximately 6–8 mya (Haas et al., 2021).

**Table 2:** Comparison of *Zizania latifolia* and *Zizania palustris* genome information.

Species	Variety/accession	Chromosome number	Genome size (Mb)	Gene number	Contig N50 (Mb)	Scaffold N50 (Mb)	Percentage of repetitive sequences (%)	References
*Z. latifolia*	HSD2	17	604.1	43 703	0.0128	0.59	37.7	Guo et al., 2015
*Z. palustris*	Itasca-C12	15	1 288.77	46 491	0.37	98.8	76	Haas et al., 2021
*Z. latifolia*	Huai’an	17	547.4	38 852	4.48	32.79	52.89	Yan et al., 2022

Given that *Z. latifolia* lacks a high-quality genome, research on the genetics and genes related to various characteristics of *Z. latifolia* remains considerably stunted compared to that of rice. *Zizania* is also a monoecious outcrosser with severe inbreeding depression, which increases the difficulty of genetic mapping research. The genome of *Z. latifolia* chloroplasts comprises typical circular double-stranded DNA molecules with genome size, overall structure, gene number, and gene sequence highly conserved with most terrestrial plant genomes (Zhang et al., 2016). The genome size is 136,501 bp, and its sequence was derived from the direct purification of chloroplast DNA (Zhang et al., 2016). Guo et al. (2015) used second-generation sequencing technology to build the *Z. latifolia* accession “HSD2” genome sequence for the first time. They performed transcriptome analysis of the molecular mechanism of the *Jiaobai* swollen culm formed following the infection of *Z. latifolia* by *U. esculenta*. Due to technical limitations and a lack of genetic linkage maps, the *Z. latifolia* genome remains relatively scattered at a scaffolding level, with a contig N50 of only 13 kb (**Table 2**). With third-generation technology, sequencing of the *Z. latifolia* genome revealed a total genome length of 545.36 Mb distributed over 17 chromosomes, accounting for 99.63%, and 300 corresponding sequences were obtained (the longest and shortest being 49.61 Mb and 17.01 Mb, respectively). In addition, the assembly of the *Z. latifolia* genome (Contig N50 = 4.48 Mb) is 343.62-fold longer than the previously completed “HSD2” genome (Contig N50 = 13kb). Through genome annotation, 289.5 Mb (52.89%) of the repetitive sequence was identified in the assembled genome, which is significantly higher than the 227.50 Mb (37.70%) reported in the previously assembled version (Yan et al., 2022).

### 4.2 Transcriptome research of *Z. latifolia*

Transcriptomics is the systematic study of global gene expression patterns to identify the molecular mechanisms of complex biological pathways and trait regulation networks (Ponting et al., 2009). These studies have revealed that the fungal pathogenicity-related genes of *U. esculenta*, as well as those associated with phytohormone biosynthesis, may cause culm enlargement of *Z. latifolia* (Wang et al., 2017; Li et al., 2021; Zhang et al., 2021). Moreover, two Cys2-His2 (C2H2) zinc finger proteins, GME3058_g and GME5963_g, from *U. esculenta* may impact fungal growth and infection at the initial stage of swollen culm formation (Zhang et al., 2021). Numerous *U. esculenta* genes related to effectors and teliospore formation have been shown to exhibit marked upregulation (Wang et al., 2020) and stage-specific expression patterns during the initial and subsequent enlargement of culm (Wang et al., 2017). For instance, Wang et al. (2020) reported that the expression of the melanin biosynthesis gene of the teliospore (T) strain is upregulated compared with that in the mycelia-teliospore (M-T) strain of *U. esculenta*; the T strain exhibits stronger pathogenicity and teliospore-forming properties than the M-T strain. Indeed, two types of gene regulatory networks contribute to the formation of different types of swollen culm (normal and grey) (Wang et al., 2020). During the process of culm swelling, many genes related to the synthesis, metabolism, and signal transduction of hormones of the host plant are stimulated and exhibit specific expression patterns. In particular, the expression of *ZlYUCCA9—*a flavin monooxygenase that serves as the key enzyme in the indole-3-acetic acid biosynthesis pathway—is markedly upregulated (Zhang et al., 2021). Moreover, host plant genes become differentially expressed before and after *U. esculenta* infection, some of which are primarily involved in plant hormone signal transduction and cell wall–loosening factors (Li et al., 2021). Although “a hormone–cell wall loosening model” was proposed to explain the symbiotic mechanism in culm enlargement (Li et al., 2021), cytokinins appear to play a more important role (Wang et al., 2017; Li et al., 2021).

### 4.3 Proteome research of *Z. latifolia*

Proteomics is the study of changes in cells, tissues, or protein composition of organisms, with the proteome functioning as the object of study (Singh et al., 2016). Isobaric tags for relative and absolute quantification (iTRAQ) have been used in recent years for high-throughput screening in quantitative proteomics and exhibit good quantitative results along with satisfactory reproducibility (Owiti et al., 2011; Chu et al., 2019). Based on the pattern of phenolic compound change during CWR germination, researchers selected the representative phases (germinated for 36 and 120 h) for further proteomics analysis of the mechanism of phenolic compound accumulation during CWR germination using iTRAQ. The results showed that the differentially expressed proteins in these two phases were primarily associated with metabolic pathways, including biosynthesis of secondary metabolites and phenylpropanoid biosynthesis (Chu et al., 2019). Other researchers performed two-dimensional electrophoresis of total proteins in CWR and rice seeds; the information obtained from peptide mass fingerprinting indicated that a glutelin precursor, caffeoyl coenzyme A O-methyltransferase, and putative bithoraxoid-like protein could provide good gene sources for improving rice seed quality (Jiang et al., 2016). *U. esculenta* evades host defenses through at least seven metabolic pathways and five biological processes to successfully reproduce in *Z. latifolia*. Observing the proteins extracted from the topmost internodal region below the apical meristematic tissue deriving from the infected and uninfected parts of *Z. latifolia* through transmission electron and fluorescence microscopy showed that *U. esculenta* hyphal morphological transitions and movement occurred both inter- and intracellularly, while sporulation occurred only intracellularly in selective cells (Jose et al., 2019). This study revealed why differentially expressed proteins in *U. esculenta* allow the inflorescence organization to be replaced by a swollen fleshy stem and why *Z. latifolia* develops resistance to infection by *U. esculenta* (Jose et al., 2019).

### 4.4 Metabolome research of *Z. latifolia* and *Z. palustris*

Plant metabolomics is the study of the relationship between gene function and phenotype through analysis of the metabolites in plant tissues during specific changes in the external environment (Oliver, 1997). Plant metabolic networks are complex and are typically classified as primary or secondary metabolisms; however, there is no clear boundary between these two types as they are closely related. Currently, ~200,000 metabolites have been found in plants (Wink, 1988). Some scientists have studied *Jiaobai* through metabolo- and other omic approaches (Luo et al., 2012; Luo et al., 2019; Bata Gouda et al., 2022). *Jiaobai* is of substantial economic value; however, its shelf-life and quality during post-harvest storage are reduced due to respiratory disorders, shell etiolation, surface browning, transpiration, and tissue hollowness (Luo et al., 2012, 2019). Therefore, researchers believe that the physiological, biochemical, and molecular processes involved in the post-harvest aging of *Jiaobai*, as well as post-harvest treatment methods to ameliorate aging and improve storage quality, are worth investigating (Bata Gouda et al., 2022; Qian et al., 2023). The mechanisms of *Jiaobai* aging during storage at 25°C were investigated using integrated methods of transcriptomics and metabolomics. The results showed that *Z. latifolia* aging is closely associated with reactive oxygen species (ROS) accumulation, ethylene biosynthesis, energy metabolism consumption caused by cell membrane degradation, and abiotic stress (Bata Gouda et al., 2022). Aging may also be weakly associated with ornithine decarboxylase, polyamine oxidase, transcription factor A, jasmonic acid-amino synthetase, coronatine insensitive protein 1, brassinosteroid insensitive 1 kinase inhibitor 1, mitogen-activated protein kinase, calmodulin, and catalase genes, as well as lower organic acid, l-alanine, and γ-linolenate contents (Bata Gouda et al., 2022). Following 1-MCP treatment, these genes, metabolism products, and enzyme activities changed, thereby delaying *Jiaobai* post-harvest aging (Bata Gouda et al., 2022). Recently, Qian et al. (2023) found that the lignification of *Jiaobai* during cold storage was regulated by respiratory burst oxidase homolog-mediated ROS signaling. These studies improved our understanding on the mechanisms of *Jiaobai* post-harvest aging and serve as important references for maintaining post-harvest quality, as well as extending the shelf life of *Jiaobai*.

Analysis and utilization of the nutritional components of wild rice are increasingly popular research topics. Enrichment analyses between CWR and NWR showed that the differential metabolites in the phenylpropanoid biosynthesis pathway were significantly enriched, with 357 metabolites forming a significant cluster, among which the relative content of 5 anthocyanin and 4 catechin derivatives differed significantly between CWR and NWR (Yan et al., 2019). In addition, the total phenolic, flavonoid, and proanthocyanidin contents in CWR were significantly higher than those in *Indica*, *Japonica*, and red rice (Yu et al., 2021). Using ultra-high performance liquid chromatography coupled to triple quadrupole mass spectrometry-based metabolomic methods, 159 flavonoids were identified in CWR and non-pigment rice, among which 78 exhibited differential expression. KEGG annotation and classification showed that the differentially expressed flavonoids were primarily associated with anthocyanin biosynthesis (Yu et al., 2021). This result is consistent with previous reports of flavonoid accumulation in pigmented rice seeds closely associated with changes in pericarp pigments (Shao et al., 2014). During CWR seed development, the content of total phenols and proanthocyanidins gradually increases. The metabolomic analysis also showed that 57 flavonoids were associated with changes in pericarp color and exhibited gradual increases (Yu et al., 2022). This study also explored the molecular basis of changes in pericarp color during CWR development. The results demonstrated novel perspectives on flavonoid biosynthesis and accumulation research in CWR, while laying the foundation for modern biotechnology to obtain grains with high flavonoid contents.

## 5 Functional verifications of important genes of *Z. latifolia* and *Z. palustris*

As a species transitioning from wildness to domestication, *Zizania* plants retain the excellent traits lost by many domesticated crops, thus providing a potential source for gene modification of varieties in modern rice breeding and an ideal natural resource for expanding and enriching breeding gene sources (Yan et al., 2022). Guo et al. (2015) and Yan et al. (2022) found that the *Z. latifolia* and rice genomes are highly collinear. Phylogenetic analysis shows that *Z. latifolia* is more closely related to *Oryza* than the other seven plants (*Brachypodium distachyon*, *Hordeum vulgare*, *Leersia perrieri*, *Sorghum bicolor*, *Setaria italica*, *Zea mays*, and *Arabidopsis thaliana*), with a differentiation time of 19.7–31 mya (Yan et al., 2022). Cross-incompatibility prohibits the direct transfer of these valuable traits to rice (Yan et al., 2022). However, researchers have successfully introduced various high-value genes of interest from *Zizania* to rice through transgenic technology, allowing for their functional verification and application (Abedinia et al., 2000; Shen et al., 2011; Qi et al., 2023). Abedinia et al. (2000) co-bombarded rice calli with high molecular weight *Z. palustris* DNA and a pGL2 plasmid encoding the selectable hygromycin resistance gene to generate transgenic plants with NWR grain characteristics for analysis. Amplified fragment length polymorphism analysis showed that this method successfully transferred DNA from large numbers of *Z. palustris* genes to rice (Abedinia et al., 2000). Following infection of rice with bacterial blight, a disease in which the vascular bundle leaves generally dry out and the non-fruiting rate increases, causing a 1000 grain weight reduction translating to a yield loss of 20–50%, even 100% in severe cases (Lee et al., 2005; Antony et al., 2010). Notably, mining and discovering previously lost beneficial genes in closely related *Zizania* and rice germplasm resources is one feasible strategy for addressing this situation (Shen et al., 2011). Researchers have designed specific primers based on homologous sequences, screened for a disease resistance gene from the *Z. latifolia* genome database, and performed alignment analyses on amplified nucleotide sequences. Results showed that *ZR1* is part of the nucleotide-binding site domains-C-terminal leucine-rich repeats resistance gene based on its similarity to the P-loop (kinase 1a), kinase 2, kinase 3a, and Gly-Leu-Pro-Leu (GLPL) conserved gene sequences (Shen et al., 2011). Over-expressed transgenic plants with significant resistance to bacterial blight PXO71 were obtained through *Agrobacterium*-mediated transfer to rice (*O. sativa* cv. Nipponbare), suggesting that transformation-competent artificial chromosome clones contain ≥ 1 bacterial blight resistance gene—*ZlBBR1* (Shen et al., 2011).

The tip of *Z. latifolia* culm enlarges into a fleshy edible gall after *U. esculenta* infection, and cytokinin plays a key role in this process. Two-component systems (TCS) connect cytokinin with transcriptional regulation receptors in the nucleus and play a significant role in many bioprocesses (He et al., 2020). Genome-wide identification and transcriptomics were performed on TCS genes in *Jiaobai* to analyze their expression during culm enlargement. The findings revealed that expression of *ZlCHK1*, *ZlRRA5*, *ZIRRA9*, *ZlRRA10*, *ZlPRR1*, and *ZlPHYA* was associated with *Jiaobai* culm swelling, among which *ARR5*, *ARR9*, and *ZlPHYA* were rapidly induced by trans-zeatin, supporting that cytokinin signal transduction plays a role in *Jiaobai* culm enlargement (He et al., 2020). The *Jiaobai* genome contains 11 chitinase genes (*ZlChi1-11*), many of which are differentially expressed under abiotic stressors such as salt, extreme temperatures, drought, and the presence of abscisic acid (Zhou et al., 2020). This study provides basic information for analyzing the role of the *Z. latifolia* chitinase gene family under abiotic stress.

The nutritional composition of CWR is greater than that of normal rice (at 0.26 ± 0.02 g), especially concerning its significantly high lysine content (at 0.62 ± 0.07 g; Zhai et al., 2000). *DHDPS* encoding the lysine biosynthetic enzyme dihydrodipocolinate synthase plays a significant role in lysine accumulation. Kong et al. (2009) cloned the *Z. latifolia DHDPS* and named it *ZlDHDPS*. The *ZlDHDPS* sequence maintains a high identity with known plant *DHDPS* in GenBank. RT-PCR analysis showed that *ZlDHDPS* expression has tissue specificity, as well as high-level expression in rapidly growing and reproductive tissues. Through genome collinearity and homology analysis, *ZlRc* (*Zla16G011250*) in *Z. latifolia* was found to be orthologous to *Rc* (*LOC_Os07g110200*) in rice (Qi et al., 2023). Functional validation of *ZlRc* was performed using subcellular localization and rice transgenes. The results show that *ZlRc* promotes phenolic compound accumulation and is located within the nucleus (Qi et al., 2023). Moreover, the pericarps of *ZlRc*-overexpressing rice are brown, while those of wild-type rice are non-pigmented (Qi et al., 2023). The total phenolic, flavonoid, and proanthocyanidin contents, antioxidant activity, as well as enzyme inhibitory effects of *ZlRc*-overexpressing rice were significantly higher than those of wild-type rice (Qi et al., 2023). These findings show that *ZlRc*-overexpression promotes phenolic compound accumulation in rice seeds and can bioaugment rice phenolic content. Through genome collinearity and homology analysis, *ZlqSH1a* (*Zla04G033720*) and *ZlqSH1b* (*Zla02G027130*) in *Z. latifolia* were found to be orthologous to *qSH1* (*LOC_Os01g62920*) in rice (Yan et al., 2022). Functional validation of *ZlqSH1a* and *ZlqSH1b* was performed using subcellular localization and rice transgenes, with the results showing that these genes are involved in regulating the development of the abscission layer and located within the nucleus. Scanning electron and laser confocal microscopy also showed that *ZlqSH1a* and *ZlqSH1b* over-expression resulted in a complete abscission layer between the grain and pedicel and significantly enhanced seed shattering following grain maturation in rice (Xie et al., 2022). Studies of gene function related to seed shattering in *Z. latifolia* have provided a foundation for reducing *Z. latifolia* seed shattering and thus accelerating its domestication.

## 6 Conclusion and perspectives

In summary, *Zizania* is a highly valuable economic crop that provides nutritious grains and vegetables for human use. Laying a strong foundation and utilization of *Zizania* is currently being done. Recently, genomes of *Z. latifolia* and *Z. palustris* have been completed, accelerating the collective understanding of the functional relationships between genes and characteristics, important physiological mechanisms, and genetic diversity of this genus. Hence, the multitude of desirable traits found in *Zizania* render it an ideal genetic resource for future molecular breeding aimed at enhancing the qualities of rice. Specifically, the domestication of wild plants is beneficial for ecological and sustainable development while providing a novel pathway for addressing food security and environmental challenges; however, current studies on *Zizania* remain insufficient, with the following research areas demanding further attention:

(1) Collection, identification, and improvement of germplasm resources of *Zizania*: We should survey, collect, preserve, and identify *Zizania* germplasm resources and establish a germplasm resource garden. Chemical (ethyl methanesulfonate) or physical (radiation) mutagenesis techniques will be used for improving the germplasm resources of *Zizania*. Referring to the domestication pathway of *Z. palustris*, domestication breeding of *Z. latifolia* will be carried out to screen plants with target traits such as reduced seed shattering, early flowering, consistent maturity, and high seed setting rate.
(2) Analysis of functional components in wild rice: We should use modern separation (solvent extraction), purification (purification of macroporous adsorption resin), and structural identification (spectral and non-spectral methods) techniques to conduct research on the separation and structural identification of bioactive substances from wild rice and accurately analyze the composition, and content of bioactive substances such as amylose, resistant starch, dietary fiber, flavonoids, saponins, anthocyanins, chlorophyll, and phytosterols in wild rice.

## Author contributions

Author contributions are conceptualization, investigation, visualization, writing- original draft preparation: NY, Z-FZ, Y-NX, and Q-QQ; Review and editing W-HL, Y-LL, YZ, H-MW, Y-FZ, Z-HY, D-PG, and QQ. All authors contributed to the article and approved the submitted version.
